# Refractory Lateral Leg Pain in a 25-Year-Old Competitive Runner: A Report of a Case of Fascial Herniation of the Peroneus Brevis With Complete Resolution After Surgical Decompression

**DOI:** 10.7759/cureus.41276

**Published:** 2023-07-02

**Authors:** Ethan Wilson, Anand Dhaliwal, Tara L Gallant, Kent P Sheridan

**Affiliations:** 1 Sports Medicine, California Northstate University College of Medicine, Elk Grove, USA; 2 Orthopedic Surgery, Sutter Medical Group, Sacramento, USA

**Keywords:** lateral fasciotomy, running injury, tibial stress fracture, leg mass, peroneus brevis incarceration, exercise-induced leg pain, lateral fascial herniation, peroneus brevis herniation

## Abstract

Fascial herniations of the leg occur when an intracompartmental leg muscle protrudes through weaknesses in the overlying fascial sheath. These fascial defects may be congenital or acquired from trauma involving penetrating injuries to the fascia. Increases in intracompartmental pressure, often resulting from muscular hypertrophy, can lead to muscular herniation through the weakened fascia. This may present as a leg mass which is often misdiagnosed as a hematoma, varicosity, or soft-tissue mass, leading to significant delays in treatment. We present a case of a peroneus brevis herniation in a 25-year-old male competitive runner with a history of a tibial stress fracture. This patient was referred to the senior author following three years of lateral leg pain worsened by activity. After confirmation of the herniation on MRI, the patient underwent a limited lateral compartment fasciotomy with complete resolution of symptoms at a six-month follow-up. This case demonstrates common pitfalls in the diagnosis of fascial herniations in refractory leg pain of runners. A comprehensive knowledge of this diagnosis and its risk factors can aid in the successful treatment of this patient cohort.

## Introduction

Exercise-induced leg pain (EILP) is defined as pain between the knee and the ankle that is increased during activity and relieved during periods of rest [1]. Runners are particularly susceptible to lower-extremity injuries. A systematic review documented a 32% one-year injury rate in long-distance runners and a 52% injury rate in marathon runners [2,3]. EILP represents a significant proportion of these injuries, most commonly resulting from tibial stress fractures, medial tibial stress syndrome, and chronic exertional compartment syndrome (CECS) [4]. Proper evaluation of EILP requires a broad differential which is often broken down into pathologies involving bones, muscles, blood vessels, nerves, and tendons [5]. Often overlooked is the possibility of fascial defects resulting in muscular herniation, which can be misdiagnosed as muscle hematomas and varicosities [6].

Muscle herniations of the leg are protrusions of muscle tissue through defects in the overlying fascial sheath [7]. They are often classified as either congenital or traumatic in nature. Congenital herniations may be attributed to general weakness in the muscular fascia or may occur at sites of nerve or vascular penetration. Traumatic hernias include direct penetrating injuries as well as closed fractures causing fascial tears [8]. Elevated intracompartmental pressure may contribute to fascial herniation, as evidenced by the finding of concurrent fascial herniation in 15-50% of patients receiving surgical management for CECS [8-10]. Elevations in intracompartmental pressure of up to 20% can occur from regular cardiovascular exercise [11].

Muscle herniations within the leg are not uncommon [12], but many go undiagnosed because they are often asymptomatic. In fact, fascial hernias are often diagnosed by dermatologists rather than orthopedic surgeons [13]. These masses can be misdiagnosed as vascular phenomena such as varicosities and hematomas [13]. Other considerations include but are not limited to, angiomas, lipomas, inclusion cysts, tumors, and muscular ruptures [14].

Symptomatic fascial herniations are less common, though may cause significant distress and impairment. Fascial herniations of the leg most commonly involve the anterior tibialis, as this muscle has the weakest fascial covering in the lower extremity [8,15]. Lateral fascial herniations, particularly peroneus brevis herniations, are encountered quite infrequently and have minimal representation in the medical literature [14]. We present a case of peroneus brevis herniation in a competitive middle-distance runner with a history of a tibial stress fracture.

## Case presentation

A 25-year-old male presented to his primary care practitioner (PCP) with a three-year history of intermittent lateral leg pain. The patient was a competitive middle-distance runner with a history of a tibial stress fracture at age 20. Two years after a complete resolution of the stress fracture, the patient gradually developed a dull, constant pain in his right lateral leg that was a 4/10 on the numerical pain rating scale at rest. Running elicited an 8/10 sharp pain radiating to his knee that persisted for 10-12 hours after finishing his workout. X-ray and MRI were performed and demonstrated resolution of his prior tibial stress fracture with no abnormal findings. For the next three years, the patient continued to run with intermittent pain, which gradually worsened with increasing mileage and improved with rest. Extensive conservative treatment including non-steroidal anti-inflammatory drugs (NSAIDs), ice, stretching, and foam roller therapy improved symptoms, but the patient continued to experience limitations in running distances. After three years, a new structural bulge was noted by the patient on his lateral leg with a marked increase in pain with activity. A vascular surgery consult was obtained for a presumptive diagnosis of a varicose vein but a trial of compression stockings failed to alleviate symptoms.

A repeat MRI was obtained with a marker on the region of interest, which revealed an interval development of a focal transfascial muscular herniation of the peroneal brevis muscle belly. The patient was then referred to the senior author, an orthopedic surgeon, who recommended a six-week trial of conservative measures, including stretching and therapeutic exercises with instructions to discontinue exercise with increased pain. After six weeks, the patient experienced no improvement in his symptoms, prompting the orthopedic surgeon to recommend surgical management.

**Figure 1:**
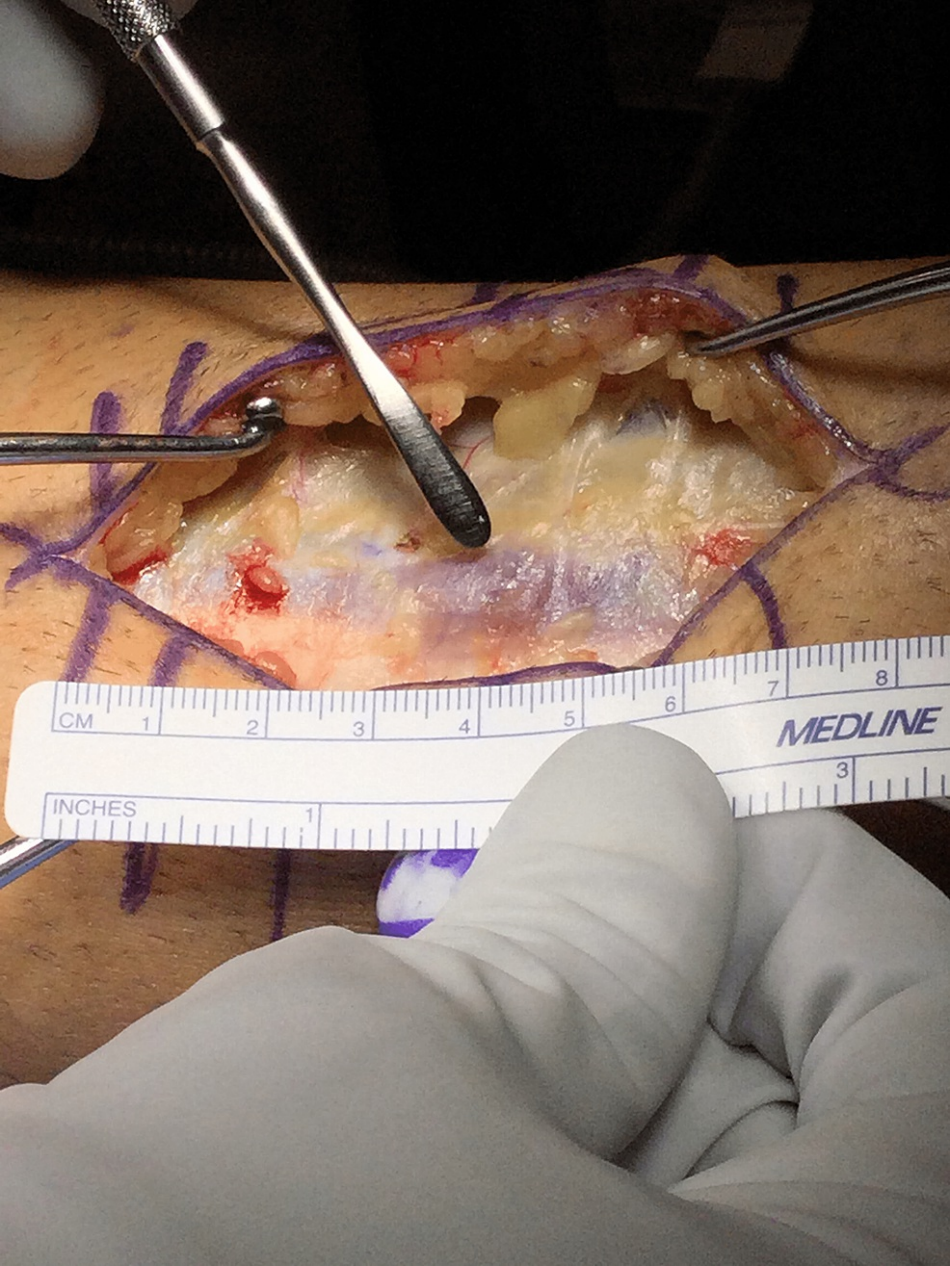
Intraoperative photo of the 2 cm fascial rent with muscular incarceration of the peroneus brevis on the right leg.

**Figure 2:**
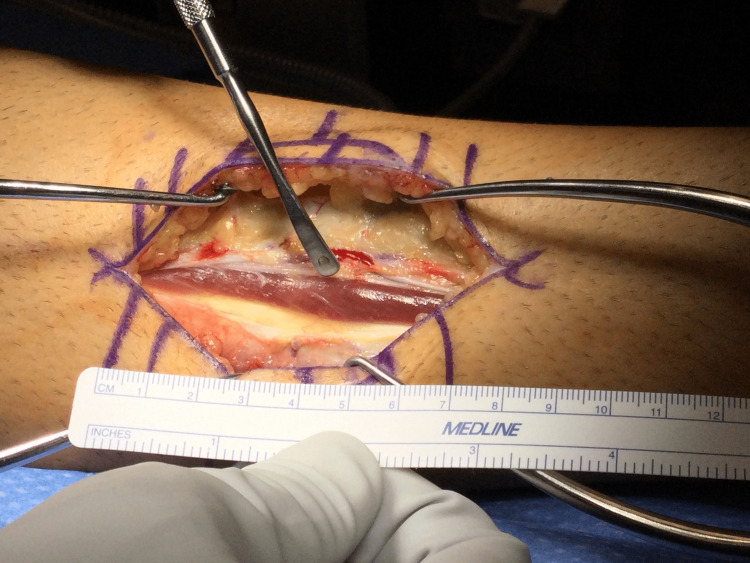
Intraoperative photo of the 6 cm longitudinal incision that was created at the site of the fascial rent.

The patient was indicated and consented to an isolated lateral compartment limited fasciotomy. A standard lateral-based approach was utilized centered over the fascial defect. The surgical technique began with identifying and marking the symptomatic region in the preoperative area that corresponded with MRI findings. The patient was positioned supine, general anesthesia was induced, and a non-sterile tourniquet was applied. A standard lateral approach utilizing a 6 cm longitudinal incision was made over the previously demarcated region. Blunt and sharp dissection was performed down to the lateral compartment fascia. The superficial peroneal nerve was identified and protected. The fascial defect was immediately apparent as a 2 cm fascial rent with muscular incarceration of the peroneus brevis (Figure 1). The rent was then sharply extended proximally and distally through the entire length of the incision to adequately decompress the incarcerated muscular tissue (Figure 2). The fascial defect was left open and the wound was irrigated and closed in layers. The skin was reapproximated with 2-0 vicryl sutures in a buried fashion and 3-0 monocryl sutures in a running fashion. A dry sterile dressing was applied and immediate weight-bearing with a gentle range of motion was allowed.

At a two-week follow-up, the wounds healed uneventfully and the patient reported significant improvement in pain to a 2/10 in severity without pain medication. The patient reported a full range of motion and denied increases in pain with walking or weight-bearing. A gradual return to normal activities, including running, was permitted with instructions to discontinue running if the pain increased. At the six-month follow-up, the patient had returned to his usual physical activities, including running without significant pain, and reported complete resolution of his pain at rest.

## Discussion

As demonstrated in the presenting case, fascial herniations are often overlooked in the differential diagnosis of leg masses [14]. This may lead to unnecessary referrals to highly impacted medical specialties such as vascular surgery and dermatology, and may subsequently lead to significant delays in care. MRI findings are often subtle and alerting the radiologist to a region of interest can aid in diagnosis [14]. It is thus important to consider fascial hernia in the differential diagnosis of leg mass, particularly in the context of EILP [1]. A more comprehensive understanding of the diagnosis of fascial herniation and possible risk factors predisposing patients to this condition could be beneficial in expediting the diagnosis and treatment of this patient cohort.

Previously documented cases of fascial herniation may provide a framework for identifying risk factors for fascial herniation. Fascial hernias can be caused by closed fractures causing direct trauma to the fascial sheath [16,17]. It is more difficult to attribute fascial herniations to tibial stress fractures, which typically have a more indolent course. However, cases of fascial herniation have occurred in patients with a history of a tibial stress fracture [18], and a previous tibial stress fracture may have predisposed this patient to subsequent fascial herniation.

Exercise-induced compartment syndrome is likely a risk factor for muscle herniations. Muscular hypertrophy secondary to cardiovascular exercise may cause significantly increased intracompartmental pressures, which can then contribute to the development of fascial hernias [11,19]. Peroneus brevis herniation has been documented in two patients with recent increases in their cardiovascular activity [14,20], and this condition most commonly affects adolescents and young adults [14,18]. The patient in this case study thus had multiple potential risk factors for fascial herniation, including age, history of a tibial stress fracture, and history of competitive running. A more thorough understanding of these risk factors may expedite and improve the treatment of this condition.

The ideal surgical management of leg muscle herniations has not been elucidated. Fasciotomy and anatomical repair with primary repair, grafting, or synthetic mesh have previously been described. Fasciotomy involves enlarging the fascial defect to relieve pain from muscular herniation and eliminate the risk of future muscular strangulation and CECS. Comparatively, anatomic repair by primary repair, grafting, or synthetic mesh is associated with an increased risk for CECS and hernia recurrence [8]. Fasciotomy was selected in this case because of the theoretical benefit of early return to function and decreased likelihood of recurrence with repair, particularly in this patient who plans on returning to extensive cardiovascular exercise.

## Conclusions

Fascial herniations are often overlooked in the differential diagnosis of both ELIP and leg masses. Consequently, it is not uncommon for these patients to experience delays in both diagnosis and treatment, as demonstrated in the presenting case. These delays can cause significant distress and functional impairment. Although the literature on fascial herniations is limited, consistent themes among patients who have suffered these injuries may provide insight into which patients are at higher risk, such as runners and those with a history of leg trauma. Improved practitioner understanding of these risk factors may expedite the management of patients with fascial herniations.
